# *Dictyostelium* Myosin Bipolar Thick Filament Formation: Importance of Charge and Specific Domains of the Myosin Rod

**DOI:** 10.1371/journal.pbio.0020356

**Published:** 2004-10-19

**Authors:** Daniel Hostetter, Sarah Rice, Sara Dean, David Altman, Peggy M McMahon, Shirley Sutton, Ashutosh Tripathy, James A Spudich

**Affiliations:** **1**Department of Biochemistry, Stanford University School of MedicineStanford, CaliforniaUnited States of America; **2**Department of Cell and Molecular Biology, Northwestern UniversityChicago, IllinoisUnited States of America; **3**UNC Macromolecular Interactions Facility, University of North CarolinaChapel Hill, North CarolinaUnited States of America

## Abstract

Myosin-II thick filament formation in *Dictyostelium* is an excellent system for investigating the phenomenon of self-assembly, as the myosin molecule itself contains all the information required to form a structure of defined size. Phosphorylation of only three threonine residues can dramatically change the assembly state of myosin-II. We show here that the C-terminal 68 kDa of the myosin-II tail (termed AD-Cterm) assembles in a regulated manner similar to full-length myosin-II and forms bipolar thick filament (BTF) structures when a green fluorescent protein (GFP) “head” is added to the N terminus. The localization of this GFP-AD-Cterm to the cleavage furrow of dividing *Dictyostelium* cells depends on assembly state, similar to full-length myosin-II. This tail fragment therefore represents a good model system for the regulated formation and localization of BTFs. By reducing regulated BTF assembly to a more manageable model system, we were able to explore determinants of myosin-II self-assembly. Our data support a model in which a globular head limits the size of a BTF, and the large-scale charge character of the AD-Cterm region is important for BTF formation. Truncation analysis of AD-Cterm tail fragments shows that assembly is delicately balanced, resulting in assembled myosin-II molecules that are poised to disassemble due to the phosphorylation of only three threonines.

## Abstract 2

A portion of the myosin tail coupled to a green fluorescent protein "head" proves a valuable model for understanding myosin self-assembly

## Introduction

### The Assembly of Bipolar Thick Filaments Is Regulated During Cell Division

**Figure 1:**
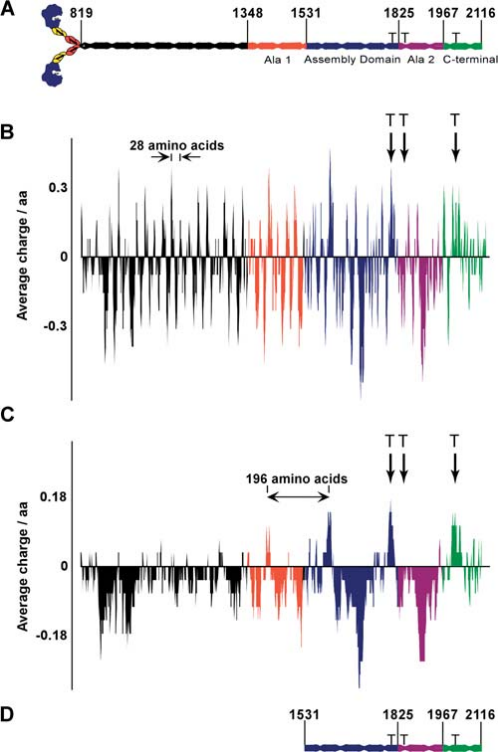
Domains and Charge Distribution within the Myosin Tail (A) The myosin head (1–818) and light chains are shown at the N terminus. In the coiled-coil tail, Ala 1 is red (1,348–1,530), the AD is blue (1,531–1,824), Ala 2 is purple (1,825–1,966), the C-terminal domain is green (1,967–2,116), and the remainder is black (819–1,347). Phospho-threonines (at positions 1,823, 1,833, and 2,029) are indicated by the letter T. (B and C) Plots of the average charge of each tail domain color coded as in (A). The y-axis is average charge; the x-axis is tail position. Aspartic acid and glutamic acid are assigned –1, lysine and arginine are assigned +1, and all other a.a. are assigned 0. The average charge in (B) was determined with a window size of 14 a.a., and the average charge in (C) was determined with a window size of 28 a.a.. Arrows highlight the 28 a.a. charge repeat in (B) and the 196 a.a. charge repeat in (C). (D) “Headless” AD-Cterm (3xThr) (1,531–2,116).

Myosin-II (hereafter referred to as myosin) is a hexameric protein composed of two heavy chains, two regulatory light chains, and two essential light chains (Figure 1A). The heavy chain consists of an N-terminal globular head that contains ATP and actin-binding sites, an α-helical neck that contains the light-chain binding sites, and finally a long α-helix that dimerizes with the α-helix of the other heavy chain to form the coiled-coil tail.

A bipolar thick filament (BTF) is a highly organized structure composed of individual myosin molecules. In vitro, myosin from the cellular slime mold Dictyostelium discoideum assembles without the aid of a cofactor, demonstrating that the myosin molecule itself contains all the information needed to form a highly organized structure of a defined size (Clarke and Spudich 1974). While proteins that co-assemble with muscle myosin have been discovered in some cell types, no such proteins have been identified in *Dictyostelium* (Barral and Epstein 1999).

In vivo, BTFs are important in a variety of cellular processes, including cell motility, chemotaxis, and development. In cytokinesis, BTFs provide the mechanical force to constrict an actin ring positioned at the midzone of the dividing cell (De la Roche et al. 2002). In *Dictyostelium,* phosphorylation of myosin inhibits filament assembly (Kuczmarski and Spudich 1980). Myosin is recruited to the cleavage furrow in the form of a BTF (Sabry et al. 1997). After the BTF provides contractile force, a myosin heavy chain kinase is recruited that then drives the disassembly of the BTF (Liang et al. 2002).

Phosphorylation of only three threonines in the 2,116-amino acid (a.a.) heavy chain of myosin is sufficient to inhibit BTF formation (Vaillancourt et al. 1988; Luck-Vielmetter et al. 1990). These threonines have been mutated to aspartic acid (3xAsp myosin) to create a mimic of a fully phosphorylated state (Egelhoff et al. 1993). 3xAsp myosin is assembly-incompetent in vitro, and the phenotype of *Dictyostelium* cells expressing only 3xAsp myosin is similar to the myosin-null mutant, consistent with previous work demonstrating that BTF assembly is required for myosin function in vivo (De Lozanne and Spudich 1987; Knecht and Loomis 1987; Manstein et al. 1989; Egelhoff et al. 1993). Mutation of the phosphorylation sites to alanines (3xAla myosin), in contrast, does not alter the filament formation properties of myosin in vitro. In vivo, 3xAla myosin is always assembled and therefore is a good mimic of an unphosphorylated state (Egelhoff et al. 1993; Yumura 2001). The doubling time of 3xAla cells is 17 h, whereas in wild-type cells it is 13 h, suggesting that thick filament disassembly is required for efficient cytokinesis (Egelhoff et al. 1993).

### The Role of Charge Repeats in the Myosin Tail

Myosin tails have a striking pattern of charged a.a., with an average positive charge over 14 a.a. followed by an average negative charge over 14 a.a. to form a 28-a.a. charge repeat throughout the tail (McLachlan and Karn 1983; McLachlan 1984). A second charge repeat found only in the C-terminal 68 kDa of the *Dictyostelium* myosin tail occurs every 196 a.a. (Warrick et al. 1986; Shoffner and De Lozanne 1996). These repeating patterns can be visualized by considering the tail as a one-dimensional rod and calculating the average charge over a window of a.a. as a function of position in the tail (Shoffner and De Lozanne 1996). A 14-a.a. window makes the 28-a.a. repeat apparent (Figure 1B), and a 28-a.a. window averages out the 28-a.a. pattern, making the 196 a.a. pattern more apparent (Figure 1C).

### The Myosin Tail Contains Functional Domains

The C-terminal 68 kDa of the tail contains the 34-kDa assembly domain (AD; a.a. 1,531–1,824; Figure 1, blue coiled-coil). The AD has salt-dependent solubility properties like full-length, wild-type myosin in vitro (O'Halloran et al. 1990; Shoffner and De Lozanne 1996). However, unlike full-length myosin, which assembles into BTFs, the AD assembles into paracrystals having undefined size and number of elements. The AD also appears to be the minimal assembling portion of the *Dictyostelium* myosin tail. C-terminal truncation of full-length myosin to a.a. 1,819 yields an assembly-competent myosin, whereas deletion of an additional 35 a.a. abolishes assembly (Lee et al. 1994).

### The Role of a Globular Head in Assembly

Replacement of the catalytic domain and essential light chain binding domain of full-length *Dictyostelium* myosin with green fluorescent protein (GFP) produces a chimera that is apparently indistinguishable from full-length myosin in its ability to form BTFs (Zang and Spudich 1998). Therefore, the myosin head is not required for BTF formation. However, the contribution of a globular head to the assembly process has not been closely examined.

### Models of Regulation

Several models have been proposed for the mechanism of myosin regulation. In this discussion, the hexameric myosin molecule will be referred to as a monomer (Figure 1A). A monomer-sequestering model was hypothesized based on the characterization of 3xAsp myosin in vitro by rotary shadowing electron microscopy (Pasternak et al. 1989; Liang et al. 1999) and in vivo by identification of myosin tail mutations that suppressed the 3xAsp phenotype (Liang et al. 1999). In this model, the coiled-coil tail bends and folds back on itself, sequestering the AD from other myosin molecules. It was further hypothesized that the bent conformation is stabilized by an intramolecular interaction between two regions of the tail rich in alanines in core positions of the heptad repeat. These coiled-coil regions, termed Ala 1 (a.a. 1,348–1,530) and Ala 2 (a.a. 1,825–1,966), are shown in red and purple respectively in Figure 1. A bias for alanines in core heptad positions is characteristic of antiparallel tetrameric coiled-coils (Munson et al. 1996), prompting the speculation that Ala 1 and Ala 2 form an antiparallel tetrameric coiled-coil in a phosphorylation-dependent manner.

An alternative monomer-sequestering mechanism might be destabilization of the coiled-coil. Threonine 1,823 is located at the C terminus of the AD, and is predicted to be in the D position of the heptad repeat. The D position is in the core of the coiled-coil where the two strands interact closely. It is possible that phosphorylation of threonine 1,823 would cause a local disruption of the coiled-coil and perturb self-assembly by introducing negative charge into the hydrophobic core (Luck-Vielmetter et al. 1990; Liang et al. 1999; Nock et al. 2000). Consistent with this hypothesis, Nock et al. (2000) showed that in full-length myosin, position 1,823 makes the largest contribution to the 3xAsp phenotype. However, the susceptibility of the myosin tail to proteolysis by chymotrypsin does not change with phosphorylation, arguing that large structural changes due to phosphorylation are unlikely (Cote and McCrea 1987).

Another model postulates that regulation occurs at the level of assembly intermediates. The assembly pathway of full-length *Dictyostelium* myosin consists of a nucleation phase followed by an elongation phase (Mahajan and Pardee 1996). In the nucleation phase, a parallel dimer forms in which two myosin monomers self-associate in a parallel orientation with a 14-nm stagger.

When two myosin monomers are offset by 14 nm, both the 28-a.a. and 196-a.a. charge repeats are aligned to maximize charge complementation between the two tails (De Lozanne 1988). Threonine 1,823 is positioned within a cluster of positively charged a.a. that forms part of the 196 a.a. repeat (Luck-Vielmetter et al. 1990; Nock et al. 2000). The introduction of negative charge by phosphorylation of threonine 1,823 could disrupt critical charge-charge interactions required for assembly.

### Localization Models

Determining the mechanism of regulated assembly will help elucidate the largely uncharacterized mechanism of myosin recruitment to the cleavage furrow. Reports that 3xAsp myosin does not localize, while 3xThr (i.e., wild-type) and 3xAla can localize, have demonstrated the necessity of BTF assembly for proper localization of myosin (Sabry et al. 1997). However, the motor activity is not required, as the chimeric GFP-regulatory light-chain tail protein is capable of translocation to the cleavage furrow in dividing *Dictyostelium* cells (Yumura and Uyeda 1997; Zang and Spudich 1998). Chimeric constructs consisting of the *Dictyostelium* motor domain and the *Acanthamoeba,* chicken smooth muscle, or skeletal muscle myosin tails translocate to the cleavage furrow of *Dictyostelium* cells despite almost no sequence homology between these tails and the *Dictyostelium* myosin tail (Shu et al. 1999; Shu et al. 2002). This has led to the hypothesis that no specific a.a. sequence is required, but that assembly is both necessary and sufficient for cleavage furrow localization (Shu et al. 1999; Shu et al. 2002; Shu et al. 2003).

### Reconstitution of Regulated BTF Assembly

Characterization of *Dictyostelium* BTFs has identified functional domains and repeating patterns in the coiled-coil tail. How do these properties come together to form a BTF that can be regulated by phosphorylation of only three a.a.? How do these properties contribute to regulated assembly? How do these properties enable a myosin BTF to localize to the cleavage furrow during cytokinesis? We have approached these questions by reconstituting regulated assembly of BTFs and defining the minimal part of the molecule required for regulated BTF assembly in vitro and in vivo.

## Results

### The AD-Cterm (3xThr) and (3xAsp) Tail Fragments Reconstitute Regulated Assembly

We wished to test whether both the Ala 1 and the Ala 2 domains of myosin are necessary to regulate BTF assembly (Liang et al. 1999) or whether the AD-through-C-terminal portion of the tail is sufficient for formation of regulated BTFs. We constructed a tail fragment that starts at the AD and extends to the end of the C terminus (AD-Cterm) (Figure 1D), and therefore contains all three threonine phosphorylation sites but not the Ala1 region. We created both “wild-type” (AD-Cterm [3xThr]) and “3xAsp” (AD-Cterm [3xAsp]) versions; the latter construct had aspartic acids in place of the three threonine phosphorylation sites to mimic a constitutively phosphorylated state. We then compared the salt-dependent solubility of these tail fragments to full-length phosphorylated and unphosphorylated myosin to see whether AD-Cterm recapitulates regulated assembly.

**Figure 2:**
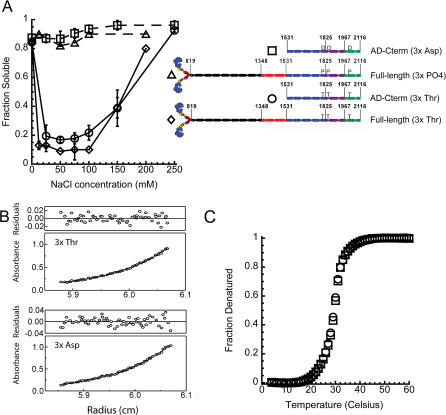
Characterization of “Headless” AD-Cterm Tail Fragments (A) Analysis of assembly by sedimentation. Fraction of soluble protein as a function of NaCl concentration is plotted for the constructs depicted adjacent to the graph. The solubility of “headless” AD-Cterm (3xThr) and AD-Cterm (3xAsp) are compared to the solubility of unphosphorylated and phosphorylated full-length myosin having the globular motor domain (full-length myosin data from Cote and McCrea [1987]). (B) Sedimentation equilibrium analysis of 52 μM “headless” AD-Cterm (3xThr) and AD-Cterm (3xAsp) in buffer containing 500 mM NaCl. The top graphs show the concentration distribution, fit, and residuals for AD-Cterm (3xThr), while the bottom graphs show the same data for AD-Cterm (3xAsp). The molecular weight obtained from the fit was 130 kDa for AD-Cterm (3xThr) and 120 kDa for AD-Cterm (3xAsp). (C) Thermal melts of “headless” AD-Cterm (3xThr) and AD-Cterm (3xAsp) in buffer containing 500 mM NaCl are shown as fraction of protein denatured as a function of temperature. The open circles are data for 50 μM “headless” AD-Cterm (3xThr), and the open squares are data for 50 μM “headless” AD-Cterm (3xAsp).

Thick filaments and paracrystals efficiently sediment upon centrifugation, whereas unassembled molecules remain soluble. Wild-type full-length myosin efficiently assembles in buffers of intermediate ionic strength (25–100 mM NaCl), while phosphorylated full-length myosin is unassembled (Figure 2A) (Kuczmarski and Spudich 1980; Cote and McCrea 1987). Therefore, regulated assembly is biochemically defined as salt-dependent insolubility of unphosphorylated myosin and solubility of phosphorylated myosin. AD-Cterm (3xThr) and AD-Cterm (3xAsp) possess the same in vitro assembly properties as full-length unphosphorylated and full-length phosphorylated myosin, respectively, suggesting that this part of the tail contains all the information needed to regulate assembly in vitro (Figure 2A).

### The Global Stabilities of AD-Cterm (3xThr) and AD-Cterm (3xAsp) Are Identical

We next tested whether AD-Cterm (3xThr) and AD-Cterm (3xAsp) tail fragments form coiled-coils, similar to full-length myosin. The oligomerization state of the tail fragments was determined by sedimentation equilibrium analysis, and the secondary structure was assessed by circular dichroism (CD). Neither tail fragment is expected to assemble in the buffers used for these studies, as they contain 500 mM NaCl. Sedimentation runs were carried out as a function of protein concentration, and equilibrium traces were obtained after 20 h of centrifugation. The profiles were fit to an equation describing the sedimentation behavior of a single, non-associating, ideal species (Figure 2B). The distribution of residuals around 0 suggests that the concentration distribution is well described by the model. The predicted molecular weight of the coiled-coil is 137 kDa, and the experimentally determined molecular weight ranged from 130 kDa to 160 kDa for the AD-Cterm (3xThr) tail fragment and 120 kDa to 150 kDa for the AD-Cterm (3xAsp) tail fragment. The range of molecular weights obtained for both tail fragments was due to an observed decrease in molecular weight as protein concentration was increased. This trend is a hallmark of non-ideality and has been observed for elongated, rod-like molecules such as DNA and skeletal muscle myosin (Tanford 1961). Far-UV CD spectra at 4 °C show that both tail fragments are α-helical with similar α-helical content as assessed by comparing mean residue ellipticity at 222 nm (θ_222_) (unpublished data). Therefore, both tail fragments behave as two-stranded coiled-coils in these assays.

The sedimentation equilibrium analysis demonstrates that a direct comparison of coiled-coil stability can be made by thermal denaturation at 500 mM NaCl because neither tail fragment self-assembles to form larger species in this condition. Thermal melts are reversible and at equilibrium and far-UV CD spectra show both tail fragments are α-helical at the starting temperature of the melt (4 °C) and are random coil at the ending temperature (60 °C) (unpublished data). The melting temperature for the AD-Cterm (3xThr) and AD-Cterm (3xAsp) tail fragments at both protein concentrations are identical (29 °C at 50 μM and 28 °C at 2.5 μM; Figure 2C). In summary, the AD-Cterm tail fragments behave as two-stranded coiled coils with similar thermal denaturation properties in high salt (Figure 2B and 2C). Therefore, the difference in assembly observed between the wild-type and 3xAsp tail fragments cannot be attributed to a failure of AD-Cterm (3xAsp) to fold into an α-helical two-stranded coiled-coil.

### AD-Cterm Tail Fragments That Have a GFP “Head” Form BTFs

**Figure 3:**
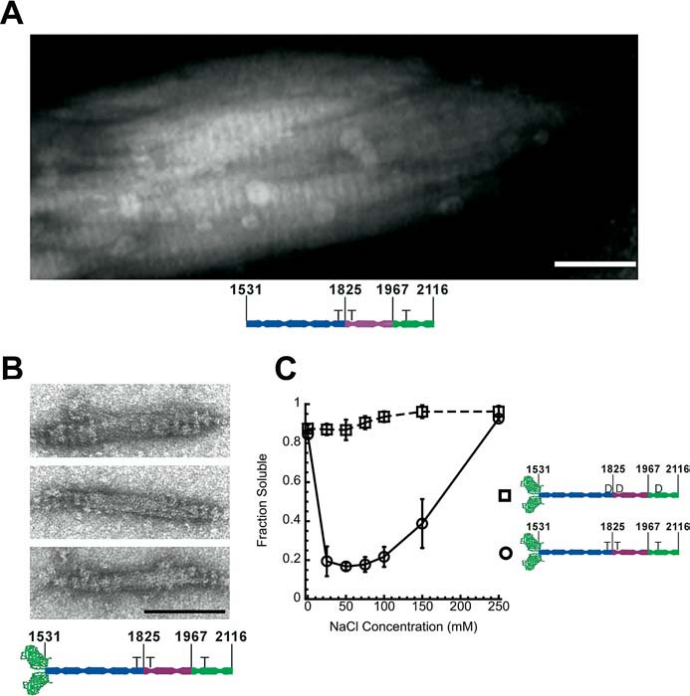
Analysis of “Headless” AD-Cterm (3xThr) and GFP-AD-Cterm (3xThr) Assembly by EM (A and B) The scale bars are 100 nm, and all panels are on the same scale. (A) The “headless” AD-Cterm (3xThr) tail fragment assembled for 2 h. (B) Three images of GFP-AD-Cterm (3xThr) assembled for 2–5 min. (C) Analysis of GFP AD-Cterm (3xThr) (open circles) and GFP-AD-Cterm (3xAsp) (open squares) assembly by sedimentation.

The AD-Cterm data show that tail fragment solubility accurately predicts the bulk solubility properties of full-length myosin. However, they do not form true BTFs. When AD-Cterm (3xThr) is assembled in buffer containing 50 mM NaCl with 10 mM MgCl_2_ and imaged using negative-stain (EM), it forms paracrystals with a 14-nm (98-a.a.) periodicity, corresponding to the periodicity of charge in the AD-through-C-terminal region of the myosin tail (De Lozanne et al. 1987; O'Halloran et al. 1990) (Figure 3A).

To test whether the presence of a globular domain might induce AD-Cterm to form regulated, bipolar structures analogous to the thick filaments formed by full-length *Dictyostelium* myosin, we attached a GFP molecule to the N terminus of AD-Cterm (GFP-AD-Cterm). Both GFP-AD-Cterm (3xThr) and GFP-AD-Cterm (3xAsp) versions of this protein were made to mimic the unphosphorylated and phosphorylated states of myosin, respectively.

We assembled purified GFP-AD-Cterm (3xThr) in buffer containing 50 mM NaCl with 10 mM MgCl_2_ and imaged them using EM. Bipolar structures first formed on a time scale of 2–5 min (Figure 3B), then bound together and reorganized to form larger, but less well-defined structures on a time scale of more than 10 min. This result is consistent with previous data on full-length *Dictyostelium* myosin indicating that when it is prepared by dialysis rather than by rapid dilution as used here (see Materials and Methods), it forms elongated structures (Stewart and Spudich 1979). The bipolar structures have striations at their ends spaced 14.4 ± 2.9 nm (*n* = 25) apart, corresponding well to the 14.3-nm offset of full-length myosin heads in a BTF (Stewart and Spudich 1979).

### GFP-AD-Cterm (3xThr) BTFs Are Structurally Homologous to Full-Length Myosin BTFs

We compared the dimensions of GFP-AD-Cterm (3xThr) and full-length myosin BTFs (Clarke and Spudich 1974; Stewart and Spudich 1979). The width of GFP-AD-Cterm (3xThr) BTFs is 27 ± 6 nm (*n* = 63) at the center of the bare zone (area where heads are absent), close to the width of full-length myosin BTFs (33 ± 1 nm). For both GFP-AD-Cterm and full-length myosin, the length of the bare zone is approximately equal to the length of the coiled-coil. This length is 130–190 nm for full-length myosin and 85 ± 11 nm (*n* = 63) for GFP-AD-Cterm. This is an indication that GFP-AD-Cterm probably assembles in a manner similar to full-length myosin, with differences in BTF dimensions reflecting differences in myosin tail length.

Notably, the solubility properties of GFP-AD-Cterm (3xThr) and GFP-AD-Cterm (3xAsp) (Figure 3C) are similar to the headless AD-Cterm (3xThr) and AD-Cterm (3xAsp) tail fragments, respectively, as well as to unphosphorylated and phosphorylated full-length myosin-II, respectively (see Figure 2A).

### The AD-Cterm Tail Fragment Is Sufficient for Regulated Localization of Myosin

To test the ability of GFP-tail fragments to localize to the cleavage furrow of *Dictyostelium* in vivo, we expressed our constructs in myosin-null *Dictyostelium* cells. However, the GFP-tail fragments were vastly overexpressed in vivo (unpublished data). Introducing the 31-a.a. regulatory light chain (RLC) binding site between the GFP and tail fragment sequences (Zang and Spudich 1998) produced expression levels similar to wild-type myosin (unpublished data). We expressed 3xThr, 3xAla, and 3xAsp versions of GFP-RLC-AD-Cterm in myosin heavy chain-null *Dictyostelium* cells. To ensure that the RLC did not interfere with filament formation, we assayed for assembly of the GFP-RLC-tail fusions in *Dictyostelium* cell extracts. As expected, the 3xThr and 3xAla GFP-RLC-AD-Cterm proteins were assembly-competent in *Dictyostelium* extracts, while GFP-RLC-AD-Cterm (3xAsp) was not (unpublished data), demonstrating that the RLC does not interfere with regulated filament formation.

**Figure 4:**
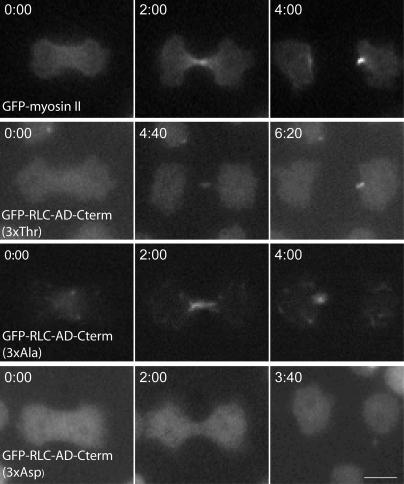
Localization of GFP-RLC-Tail Fragment Constructs in Live Dividing *Dictyostelium* Cells The localization of several GFP-RLC-myosin tail fragments during and just after cytokinesis in live *Dictyostelium* cells are shown. GFP-myosin (row 1) is clearly localized to the early and late cleavage furrow of the dividing cell and to the back end of the resulting daughter cells. By contrast, GFP-RLC-AD-Cterm (3xThr) (row 2) is localized correctly only at the late stages of cytokinesis and in the back end of one daughter cell. GFP-RLC-AD-Cterm (3xAla) (row 3) is localized to the furrow as well as to the back end of a daughter cell, while GFP-RLC-AD-Cterm (3xAsp) (row 4) shows diffuse localization throughout cytokinesis. The scale bar is 10 μm and the time is indicated in min:sec.

We used live-cell fluorescence microscopy to study the localization of the GFP-RLC-tail fragments in *Dictyostelium* cells (Figure 4). In 9 of 9 dividing cells, GFP-RLC-AD-Cterm (3xAla) localized to the cleavage furrow and remained at the site of cleavage furrow formation in the resulting daughter cells (termed “back end”) after cytokinesis. In contrast, GFP-RLC-AD-Cterm (3xAsp) did not go to the cleavage furrow in any of 11 observed dividing cells. GFP-RLC-AD-Cterm (3xThr) localized to the furrow in 6 of 12 dividing cells, and in these six cells localization was apparent only in the very late stage of furrow formation and at the back end of the resulting daughter cells. In three cells, GFP-RLC-AD-Cterm (3xThr) localization was clearly visible only at the back end of the resulting daughter cells, and in the remaining three cells no localization was clear. Thus, the AD-Cterm tail fragment is sufficient for localization, but does not localize as efficiently as full-length GFP-myosin (3xThr). The increased localization of GFP-RLC-AD-Cterm (3xAla) is consistent with the overassembly and increased localization reported for full-length GFP-myosin (3xAla) (Sabry et al. 1997; Robinson et al. 2002).

### Objects of Comparable Length to BTFs Are Not Enriched at the Cleavage Furrow During Cytokinesis

**Figure 5:**
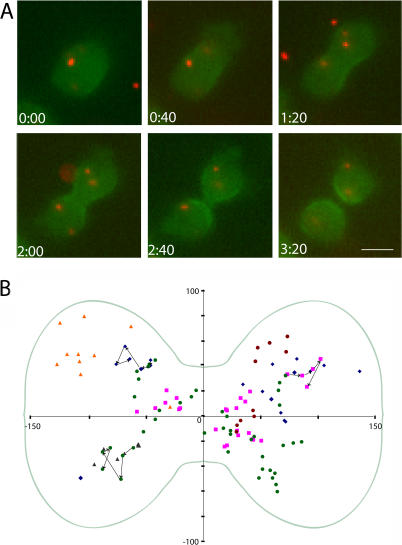
Localization of 0.5-μm Beads in Live Dividing *Dictyostelium* Cells (A) Time course of a representative GMO8B *Dictyostelium* cell (defined in Materials and Methods; contains GFP myosin) during cytokinesis. GFP-myosin fluorescence is shown in green and the beads are shown in red. While GFP-myosin accumulates in the furrow, the beads show no directed motion. The scale bar is 10 μm and time is indicated in min:sec. (B) Plot of the location of each bead in each of six dividing cells in the six frames imaged during cytokinesis. The axes were defined in each frame to bisect the center of the cell both horizontally and vertically such that the center of the furrow is the origin of the axes. The position of each bead was then plotted relative to these axes. Different beads are represented by different colors. For three of the beads, the trajectory of the bead is shown with arrows. The average location of a cell is outlined on the plot in transparent green.

To examine the specificity of myosin BTF localization, we examined whether any object of comparable length to a BTF is enriched at the cleavage furrow during cytokinesis (Uyeda and Yumura 2000). We scrape-loaded 0.5 μm diameter fluorescent beads into *Dictyostelium* cells expressing GFP fused to the N terminus of full-length wild-type myosin. These are round beads, while *Dictyostelium* BTFs are rod-shaped, but the length of each structure is comparable (approximately 0.5 μm). Figure 5A shows a time course of a representative *Dictyostelium* cell during cytokinesis. In these cells, GFP-myosin is recruited to the cell equator early during cytokinesis and remains there until the cell divides. Thereafter, myosin remains at the back end of the daughter cell as they move away from one another. In contrast, the labeled beads of similar size show no directed motion toward the furrow (Figure 5B).

### Are All the Domains within the AD-Cterm Tail Fragment Required for Regulated Assembly

**Figure 6:**
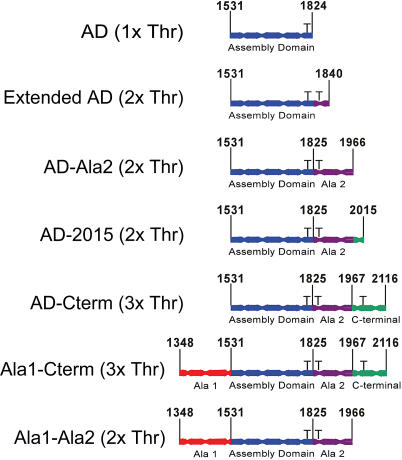
Tail Fragments Used for Truncation Analysis For tail fragments that include more than one domain, the name is determined by the first and last domain in the tail fragment. Phosphorylation sites are indicated in parentheses. 1xThr indicates that the fragment contains the threonine at a.a. 1,823; 2xThr indicates that the fragment contains the threonines at a.a. 1,823 and 1,833; and 3xThr indicates that the fragment contains the threonines at a.a. 1,823, 1,833, and 2,029. The same scheme is used to describe aspartic acid-containing constructs in the paper, except threonine is substituted with aspartic acid.

The data detailed in Figures 2–4 show that the tail fragments examined in this study are a good model for a myosin BTF. To examine the roles of the various domains within this tail fragment, we have constructed several shorter fragments of the myosin tail (Figure 6) and analyzed their regulated assembly properties.

### The AD Does Not Reconstitute Regulated Assembly

**Figure 7:**
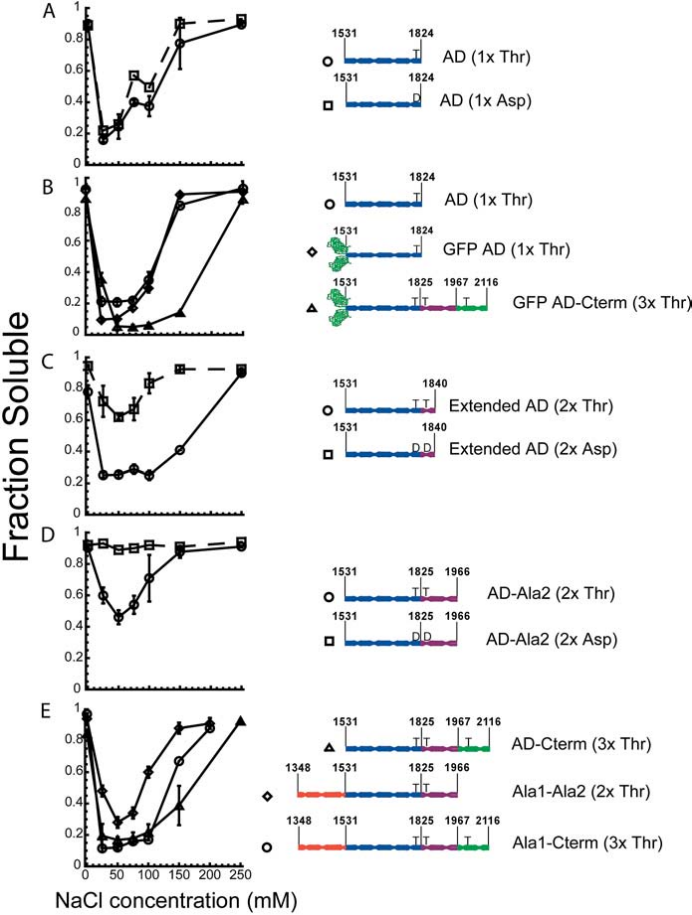
Analysis of Assembly by Sedimentation The solubility of the various constructs used for the truncation analysis is compared. (A) Comparison of “headless” AD (1xThr) and “headless” AD (1xAsp). (B) Comparison of “headless” AD (1xThr), GFP-AD (1xThr), and GFP-AD-Cterm (3xThr). The GFP is located at the N-terminus in both GFP-containing constructs. (C) Comparison of “headless” extended AD (2xThr) and “headless” extended AD (2xAsp). (D) Comparison of “headless” AD-Ala2 (2xThr) and “headless” AD-Ala2 (2xAsp). (E) Comparison of “headless” AD-Cterm (3xThr), “headless” Ala1-Ala2 (2xThr), and “headless” Ala1-Cterm (3xThr).

Given the large contribution that a.a. 1,823, the penultimate a.a. in the AD, makes to the 3xAsp phenotype (Nock et al. 2000), we tested whether the AD could be regulated by this a.a. alone. The salt-dependent solubilities of the AD (1xThr) and a mutant in which threonine 1,823 had been changed to aspartic acid (AD [1xAsp]) were nearly identical in the sedimentation assay (Figure 7A), which suggests that the AD can assemble but is not sufficient for regulated assembly.

We attached a GFP “head” to the AD (1xThr) and, interestingly, this GFP-AD (1xThr) tail fragment had bulk sedimentation properties very similar to those of headless AD (1xThr), suggesting that the presence of a globular domain does not affect in vitro solubility, similar to AD-Cterm and GFP-AD-Cterm tail fragments (Figure 7B). The AD (1xThr) tail fragments are more salt-sensitive than AD-Cterm (3xThr) tail fragments, possibly because they have a shorter coiled-coil tail (Figure 7A and 7B). EM showed that GFP-AD (1xThr) formed structures of fixed size that were not true BTFs because they did not contain bare zones (unpublished data). This demonstrates that a globular head fixes the size of assembling myosin structures, while C-terminal sequence elements in the myosin tail may be required for proper formation of the bare zone.

### Regulated Assembly Cannot Be Reconstituted with Tail Fragments Smaller Than AD-Cterm

To determine whether the entire 34-kDa portion of the tail C-terminal to the AD is required for regulated assembly, we generated a series of tail fragments that all start at the AD and are truncated at different positions within the C-terminal 34 kDa of the tail (see Figure 6 for constructs). The first such fragments that we examined started at the AD and included threonine 1,833, the second phosphorylation site (extended AD [2xThr]) and extended AD [2xAsp]). Unlike AD (1xThr) and AD (1xAsp), extended AD (2xThr) and extended AD (2xAsp) show a four-fold difference in solubility (Figure 7C). This is significantly less than the eight-fold difference in solubility of full-length unphosphorylated and phosphorylated myosin, respectively, and AD-Cterm (3xThr) and AD-Cterm (3xAsp), respectively.

To test whether adding on a larger portion of the tail results in a greater degree of regulated assembly, we constructed tail fragments that start at the AD and end at a.a. 1,966, the end of Ala 2 (AD-Ala 2). As with other tail fragments, the aspartic acid variant of this construct is more soluble than its wild-type counterpart (Figure 7D). Surprisingly, AD-Ala 2 (2xThr) exhibits inhibited assembly relative to shorter constructs (Figure 7D). This inhibition was not merely a consequence of where we truncated this tail fragment. A tail fragment was constructed that started at the AD and ended at a.a. 2,015 (AD-2015 [2xThr]). This construct contains an isoleucine in the C-terminal most heptad core position, making it less likely that the two strands of the coiled-coil will fray. Like AD-Ala 2 (2xThr), AD-2015 (2xThr) exhibits inhibited self-assembly (unpublished data). Furthermore, a far-UV CD spectrum of AD-Ala2 (2xThr) showed that it is α-helical (unpublished data), indicating that AD-Ala2 (2xThr) is structurally intact. All tail fragments truncated in the C-terminal 34 kDa of the tail exhibit altered assembly properties, while AD-Cterm (3xThr) and AD-Cterm (3xAsp) possess the same in vitro assembly properties as full-length, unphosphorylated, and phosphorylated myosin, respectively (see Figure 7A) (Cote and McCrea 1987). These data suggest that the entire AD-Cterm tail fragment is required to reconstitute regulated assembly of BTFs.

### The Ala 1 Domain Stabilizes Assembly

When the entire C-terminal domain is included in the AD-Cterm tail fragment, the inhibitory effect of Ala 2 is overcome. To test whether this stabilizing effect is specific to the C-terminal domain, we determined if Ala 1 could also stabilize assembly. A tail fragment was constructed that starts at Ala 1 and ends at Ala 2 (Ala1-Ala2 [2xThr]). The salt-dependent assembly of Ala1-Ala2 (2xThr) is more efficient than that of AD-Ala2 (2xThr) (Figure 7E) at 5 μM protein. Thus, Ala 1 can partially overcome the inhibitory effect of Ala 2, but not as robustly as the C-terminal domain. A tail fragment containing both Ala 1 and the C-terminal domain (Ala1-Cterm [3xThr]) assembles essentially the same as AD-Cterm (3xThr) (Figure 7E). All of these data indicate that the assembly reaction is delicately balanced, because the various domains of the tail make both favorable and unfavorable contributions to assembly.

### A Globular Head Need Not Be Located N-Terminal to the AD to Promote Assembly of Fixed-Size Structures

**Figure 8:**
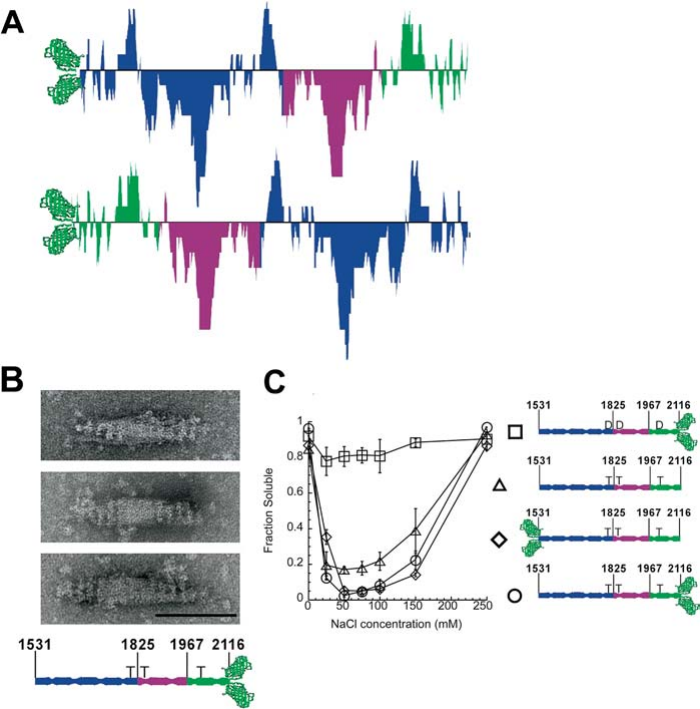
Design and Assembly Characteristics of AD-Cterm-GFP (A) The AD-Cterm charge distribution (top) is aligned with the reverse charge distribution (bottom), showing the overall symmetry of the 196-a.a. charge repeat in this region of the tail. (B) Analysis of assembly by EM. The AD-Cterm (3xThr) tail fragment has GFP on the C-terminus (AD-Cterm-GFP [3xThr]). The scale bar indicates a distance of 100 nm. Shown are three images of AD-Cterm GFP (3xThr), assembled 2–5 min. (C) Analysis of assembly by sedimentation. The solubility of AD-Cterm-GFP (3xThr) and AD-Cterm-GFP (3xAsp) tail fragments constructs are compared to GFP-AD-Cterm (3xThr) and “headless” AD-Cterm (3xThr) tail fragments.

The 196-a.a. repeat is remarkably symmetric in the entire AD-Cterm region (Figure 8A). We created a protein identical to GFP-AD-Cterm (3xThr) except that the GFP is located at the C-terminus, after a.a. 2,116 (AD-Cterm-GFP [3xThr]) (Figure 8B). Because of the symmetry in this region of the myosin tail, the AD-Cterm-GFP (3xThr) and GFP-AD-Cterm (3xThr) proteins can distinguish between assembly mechanisms that require specific a.a. in specific positions relative to the globular head versus assembly mechanisms that require a very general charge pattern to occur. AD-Cterm-GFP (3xAsp) was also created to test whether the same three threonines can regulate assembly of this myosin tail fragment.

### AD-Cterm GFP (3xThr) Assembles into Bipolar Structures

To test whether AD-Cterm-GFP (3xThr) can form BTFs, we performed EM (Figure 8B) and sedimentation assays (Figure 8C). Similar to GFP-AD-Cterm (3xThr), AD-Cterm-GFP (3xThr) formed clustered or larger structures when protein was assembled for more than 10 min prior to imaging, whereas BTFs were most prevalent when protein was assembled for 2–5 min. The striations at the ends of the AD-Cterm-GFP (3xThr) bipolar structures are spaced 14 ± 2 nm (*n* = 118) apart, consistent with the 14-nm spacing of full-length *Dictyostelium* myosin. The AD-Cterm-GFP (3xThr) structures have a shorter bare zone than GFP-AD-Cterm (3xThr) structures (62 ± 8 [*n* = 75] versus 85 nm), but a similar width to both GFP-AD-Cterm (3xThr) and full-length myosin BTFs (32 ± 5 nm [*n* = 159] versus 27 nm and 33 nm, respectively). While the AD-Cterm-GFP (3xThr) structures differ slightly from those formed by full-length myosin and GFP-AD-Cterm (3xThr), it is clear that the presence of the globular domain leads to a fixed length, even in C-terminally capped myosin tail fragments.

### AD-Cterm GFP Assembly Is Regulated

We next tested whether AD-Cterm-GFP assembly is regulated. AD-Cterm-GFP (3xAsp) is soluble at all NaCl concentrations, whereas AD-Cterm-GFP (3xThr) sediments efficiently between 25 mM and 150 mM NaCl (Figure 8C), very similar to “headless” AD-Cterm and full-length myosin (Figure 2A) as well as GFP-AD-Cterm (3xThr) (Figure 3C). Therefore, regulation of assembly does not require a specific distance between the threonine phosphorylation sites and a globular head.

## Discussion

### Regulation of AD-Cterm and Full-Length Myosin Is Identical In Vitro

The assembly properties of both AD-Cterm (3xThr) and AD-Cterm (3xAsp) closely parallel those of full-length unphosphorylated and phosphorylated myosin described by Cote and McCrea (1987). These data represent a good basis for comparison to AD-Cterm, because the myosin was heavily phosphorylated (2:1 stoichiometry of phosphate:myosin heavy chain), and Cote and McCrea performed a gel filtration step to eliminate any contaminating actin. Other sedimentation data for full-length myosin compare favorably with the AD-Cterm data as well (Kuczmarski and Spudich 1980; Egelhoff et al. 1993). The parallel between the solubility of AD-Cterm and full-length myosin in vitro argues that AD-Cterm (3xAsp) is a good mimic of the phosphorylated state of full-length myosin and that all of the information necessary for regulated assembly is present in the AD-Cterm region of the myosin tail.

### Attaching a Globular Head to AD-Cterm Reconstitutes Regulated BTF Assembly

The fact that GFP-AD-Cterm (3xThr) forms BTFs whereas AD-Cterm (3xThr) does not, demonstrates that the mere presence of a globular head differentiates BTFs from paracrystals, and that neither the myosin head nor the S2 region of the myosin coiled-coil is critical for BTF formation (Zang and Spudich 1998; this study). While the presence of the globular head is critical for the size and shape of myosin structures in this study, it has no appreciable effect on the regulation of myosin BTF assembly. Furthermore, other cellular factors, with the exception of kinases and phosphatases, are not required for BTF formation, because the process can be completely reconstituted using recombinant, bacterially expressed protein.

### Regulation of GFP-AD-Cterm and Full-Length Myosin Is Similar In Vivo

GFP-RLC-AD-Cterm tail fragments demonstrate regulated recruitment to the cleavage furrow during cytokinesis. While the localization of GFP-RLC-AD-Cterm (3xThr) is less robust than full-length myosin, constitutively assembled GFP-RLC-AD-Cterm (3xAla) always localizes to the cleavage furrow in cytokinesis (see Figure 4). In contrast, GFP-RLC-AD-Cterm (3xAsp) fails to localize (see Figure 4). These data are consistent with other observations (Sabry et al. 1997; Shu et al. 2003) indicating that assembled myosin constructs localize to the cleavage furrow while unassembled myosin constructs do not.

The difference in localization efficiency between full-length myosin and GFP-RLC-AD-Cterm (3xThr) might be due to differences in degree of assembly. The critical concentration of full-length myosin is estimated to be less than 20 nM (Mahajan and Pardee 1996). One might expect the critical concentration of assembly for GFP-RLC-AD-Cterm (3xThr) to be higher than full-length myosin, because full-length myosin has a longer tail. Likewise, the critical concentration of GFP-RLC-AD (1xThr) is expected to be higher than that of GFP-RLC-AD-Cterm (3xThr). This argument may explain why GFP-RLC-AD-Cterm (3xThr) recruitment is less efficient than full-length myosin, and why GFP-RLC-AD (1xThr) does not go to the furrow at all (unpublished data).

Interestingly, beads that are approximately the same length as the BTFs formed from full-length myosin do not localize to the cleavage furrow. Together with the previous in vivo localization data, this result argues that the majority of the information necessary for localization of BTFs to the cleavage furrow is in the AD-Cterm portion of the myosin tail, and that localization to the cleavage furrow is an active process, possibly involving another cellular factor that recruits myosin. It may be that this factor recognizes the charge repeats that distinguish assembled myosin BTFs from unassembled myosin. Consistent with this model is the localization of chimeric myosin molecules to the cleavage furrow in *Dictyostelium* cells (Shu et al. 1999; Shu et al. 2002). These chimeras have myosin tails from other species with no sequence homology to the *Dictyostelium* tail, but contain the charge repeats common to all myosin tails.

### Regulation Does Not Require an Ala 1-Ala 2 Intramolecular Interaction

Our finding that the in vitro self-assembly of AD-Cterm (3xThr) and AD-Cterm (3xAsp) are very similar to full-length, wild-type myosin and full-length, phosphorylated myosin, respectively, show that Ala 1 is not required for regulated assembly. This result has been confirmed in vivo by deleting Ala 1 from both full-length wild-type and 3xAsp myosin (W. Liang and JAS, unpublished data). *Dictyostelium* cells expressing the Ala 1 deletions as their sole source of myosin were phenotypically indistinguishable from cells expressing their full-length counterparts. It is possible that an Ala 1-Ala 2 intramolecular interaction is indeed one mode of regulation, but is not necessary, because redundant regulatory mechanisms occur at multiple points along the assembly pathway.

### Regulation Does Not Occur by Conformational Disruption of the Coiled-Coil

The CD and analytical ultracentrifugation data show that AD-Cterm (3xAsp) has not simply failed to fold into a two-stranded α-helical coiled-coil. These data are consistent with rotary shadowed electron micrographs of full-length 3xAsp and phosphorylated myosin. Gross destabilization of the coiled-coil would result in a single-headed myosin, and none have been observed (Pasternak et al. 1989; Liang et al. 1999). Global destabilization might result in a section of the tail unfolding, lowering resistance to thermal denaturation. However, thermal melts show that the melting temperature of AD-Cterm (3xThr) and AD-Cterm (3xAsp) are identical (see Figure 2C). Local destabilization of the coiled-coil seems unlikely, because one of the chymotrypsin cleavage sites is close to threonine 1,823, which is in a core position of the heptad repeat, yet the proteolytic susceptibility of the myosin tail does not change upon phosphorylation (Cote and McCrea 1987).

Small numbers of assembled AD-Cterm (3xAsp) paracrystals were seen in electron micrographs of samples (unpublished data). These paracrystals, although quite rare, possess a 14-nm periodicity similar to the wild-type tail fragment. Consistent with this observation, approximately 10% of 3xAsp myosin sediments at 50 mM NaCl, demonstrating that regulation of assembly is not an all-or-none process. Together with the CD data, this result indicates that AD-Cterm (3xAsp) is structurally and functionally intact. Rather than being conformationally disrupted, the 3xAsp tail fragment fails to assemble because the critical concentration for assembly is higher than that of the wild-type tail fragment.

### Regulation of Assembly by Modulation of Charge-Charge Interactions

#### The role of the 196 a.a. charge repeat in assembly

The fact that AD-Cterm-GFP forms BTF-like structures with heads on the outside and tails in the center demonstrates the role of the 196-a.a. charge repeat in assembly. This charge repeat is symmetric in the AD-Cterm region of the tail (see Figure 8A). If the 196 a.a. charge repeat is a major driving force of assembly, then BTF formation should be independent of whether a globular head is positioned on the N or C terminus of AD-Cterm. Since globular heads are clearly visible on the outer edges and not in the center of AD-Cterm-GFP (3xThr) structures, local interactions between a.a. in these filaments must be different than local interactions in filaments formed from GFP-AD-Cterm (3xThr) and from full-length myosin. Further structural characterization is required for a model that describes the packing of the coiled-coils within these filaments, but these data are consistent with the overall large scale charge character of the tail being important for forming BTFs.

Interestingly, AD-Cterm-GFP (3xThr) structures are shorter than GFP-AD-Cterm (3xThr) filaments, and the bare zones are smaller as well. This may be because C-terminal sequence elements in the myosin tail may be required for proper formation of the bare zone and thereby determine the exact morphology of the BTF.

#### The assembly reaction is delicately balanced.

The truncation analysis provides evidence of a delicate balance between forces that drive assembly and disassembly of myosin tails. This is most strikingly demonstrated by the observation that Ala 2 was actually found to inhibit the assembly of AD, while Ala 1 and the C-terminal domain help to drive assembly. We propose that that this delicate balance is related to the 196-a.a. charge repeat in the tail. The attachment of Ala 2 (purple, Figure 1) to the AD (blue, Figure 1) adds a large cluster of negative charge onto the end of this tail fragment. Assembly might be inhibited because a proper balance of charge is required for efficient self-assembly. This balance is restored by addition of either the C-terminal portion of the tail (green, Figure 1) or Ala 1 (red, Figure 1), because both of these domains possess clusters of positive charge.

Because the charge repeats likely play a role in each step in the assembly pathway, a small effect, such as the introduction of negative charge at key points in the molecule, could have a large effect on overall self-assembly. The threonine phosphorylation sites are positioned near the positive clusters of charge at the end of AD and in the C-terminal domain, suggesting that regulation by phosphorylation might have its largest effect on this charge pattern.

## Materials and Methods

### 

#### Construction of myosin tail fragments for expression in *E. coli*

The polymerase chain reaction (PCR) was used to amplify the regions of a *Dictyostelium* expression vector (pBIG) containing either GFP (3xThr) myosin or GFP (3xAsp) myosin (Sabry et al. 1997). An NdeI site was engineered at the 5′ end, and a stop codon followed by a SacI site was placed at the 3′ end of the PCR product. PCR products were directionally subcloned into the pET21a vector (Novagen, Madison, Wisconsin, United States) using these two restriction sites.

Tail fragments contain an N-terminal methionine followed by these a.a. from *Dictyostelium* myosin: AD (1xThr and 1xAsp), a.a. 1,531–1,824; AD-Cterm (3xThr and 3xAsp), a.a. 1,531–2,116; extended AD (2xThr and 2xAsp), a.a. 1,531–1,840; AD-Ala 2 (2xThr and 2xAsp), a.a. 1,531–1,966; AD-2015 (2xThr), a.a. 1,531–2,015; Ala 1-Ala 2 (2xThr), a.a. 1,348–1,966; Ala 1-Cterm (3xThr), a.a. 1,348–2,116 (see Figure 6). The same set of PCR primers were used to construct the wild-type and aspartic acid variants of each tail fragment except for the AD (1xAsp) fragment. For AD (1xAsp), Quikchange site-directed mutagenesis (Stratagene, La Jolla, California, United States) was used to mutate threonine 1,823 to aspartic acid. The sequence of all constructs was confirmed.

#### Construction of GFP tail fragments for expression in *E. coli*

The constructs described above are the source of tail fragment DNA for all GFP constructions. To create N-terminal fusions, 5′ and 3′ ends of GFP-UV (Crameri et al. 1996) were modified using PCR with primers that contain an NcoI site followed by a 6xhistidine tag at the 5′ end and an NdeI site at the 3′ end before the GFP-UV stop codon. Internal NdeI and NcoI sites were eliminated from the GFP-UV gene by Quikchange mutagenesis (Stratagene), and the modified GFP-UV gene was subcloned into the pET28a vector (Novagen) using NcoI and NdeI (GFP-UV-pET28a). Tail fragments were subcloned into GFP-UV-pET28a using NdeI and NotI.

To create C-terminal GFP fusions, the 5′ and 3′ ends of GFP-UV in the GFP-UV-pET28a vector were modified by PCR. The 5′ primer eliminated the N-terminal 6xhistidine tag and introduced a SacI site. The 3′ primer eliminated an internal SacI site and introduced a 6xhistidine tag, followed by a stop codon, followed by a NotI site. Quikchange mutagenesis (Stratagene) was used to eliminate the stop codon immediately before the SacI site in the AD-Cterm tail fragments contained in pET21a. The modified GFP-UV was subcloned into this vector using SacI and NotI. All DNA sequences were verified.

#### Construction of GFP tail fragments for localization studies in *Dictyostelium*

To make N-terminal GFP-tail constructs for expression in *Dictyostelium,* the myosin regulatory light chain binding site (RLCBS) sequence was ligated into plasmid pTX-GFP (Levi et al. 2000) to create pTX-GFP-RLCBS. The GFP sequence from pTX-GFP was subcloned into pET28a (Novagen) using NcoI and SacI. The NdeI site was removed from the GFP sequence using Quikchange mutagenesis (Stratagene) and then subcloned back into pTX-GFP to create pTX-GFPΔNde1. The RLCBS was amplified from pBigGFPRLC+ (Zang and Spudich 1998) using primers to add a 5′ SacI site and 3′ NdeI and XhoI sites and ligated into pTX-GFPΔNde1 using SacI and XhoI digestion. This vector is called pTX-GFP-RLCBS. Myosin tail fragments were subcloned into pTX-GFP-RLCBS using the NdeI and XhoI sites from the corresponding pET21a-tail fragment vectors. All DNA sequences were verified.

#### Protein expression in *E. coli*

All tail fragment constructs were transformed into the BL21-CodonPlus (DE3)-RIL strain (Stratagene). LB media contained 34 μg/ml chloramphenicol and either 100 μg/ml kanamycin for pET28a or 100 μg/ml carbenicillin for pET21a. Cells were grown at 37 °C to an absorbance at 600 nm of approximately 0.6. Protein expression was induced by adding 1 mM IPTG and incubating for 1 h. Cells were harvested by centrifugation at 6,370 × g for 15 min.

#### Purification of myosin tail fragments from *E. coli*

Harvested cells were resuspended in 10 mM Tris (pH 7.4), 1 mM EDTA, 1 mM DTT, 500 mM NaCl, 30% sucrose, and protease inhibitor (PI) cocktail (final concentration of 0.7 μg/ml leupeptin, 0.7 μg/ml pepstatin A, 2 μg/ml aprotinin, and 1 mM PMSF). For a single protein prep, the pellet from 10 l of culture was resuspended in buffer for a final volume of 40 ml. Cells were added dropwise into liquid nitrogen and stored at –80 °C.

Cells were thawed and 5 μg/ml RNase A (#78020Y; USB, Cleveland, Ohio, United States), 50 μg/ml RNase-free DNase I (#776 785; Roche, Basel, Switzerland), and 10 mM MgCl_2_ were added. The cells were lysed with two passes through a French Press (American Instrument Company, Silver Spring, Maryland, United States) at 10,000 pounds per square inch. After the first pass, a new batch of the PI cocktail was added. The lysate was centrifuged at 100,000 × g for 30 min at 4 °C. The supernatant was boiled for 10 min, and then a new batch of the PI cocktail and 1 mM DTT was added. The boiled supernatant was centrifuged at 100,000 × g for 30 min at 4 °C and then dialyzed against DEAE Low-Salt Buffer (DEAE-LSB; 10 mM Tris [pH 8.0], 1 mM EDTA, 1 mM DTT, and PI cocktail). The protein was injected onto a column consisting of eight tandem HiTrap DEAE Sepharose Fast Flow columns (Amersham Biosciences, Little Chalfont, United Kingdom). The column was washed with 5 volumes of DEAE-LSB and protein was eluted on a linear gradient from 0 to 500 mM NaCl over 10 volumes. Fractions containing protein (typically eluting at 230 mM NaCl) were identified by SDS-PAGE. Protein was precipitated by addition of 85% (NH_4_)_2_SO_4_, stirred at 4 °C for 30 min, and centrifuged at 100,000 × g for 30 min at 4 °C. The pellet was resuspended in a minimal volume of gel filtration buffer (10 mM Tris [pH 8.0], 1 mM EDTA, 1 mM DTT, and 500 mM NaCl) and loaded on a HiLoad 26/60 Superdex 200 column (Amersham Biosciences). Fractions were analyzed by SDS-PAGE, and those containing pure tail fragments were pooled and dialyzed against Mono-Q Low Salt Buffer (Mono-Q LSB; 10 mM Tris [pH 8], 1 mM EDTA). Protein was injected onto a Mono Q HR 5/5 column (Amersham Biosciences) and a gradient from 0 to 1 M NaCl was run. Tail fragments typically eluted at 450 mM NaCl.

Protein concentration was determined by measuring the absorbance at 280 nm in 6M guanidine hydrochloride. The extinction coefficient was calculated using the sequence of one strand of the coiled-coil as described in Gill and von Hippel (1989).

#### Purification of GFP tail fragments from *E. coli*

After harvesting, cells were resuspended in 50 mM sodium phosphate (pH 8.0) with 250 mM NaCl and containing PI cocktail. For a single protein prep, the pellet from 30 l of culture was resuspended in buffer for a final volume of 120 ml.

Lysis and the first centrifugation step were identical to the myosin tail fragment protocol described above except that 5 mM MgCl_2_ was added along with the DNase I and RNase A. The supernatant was batch-bound to Ni-NTA resin (Qiagen, Valencia, California, United States) at 4 °C for 1 h. The resin was washed with 40 ml of 50 mM sodium phosphate (pH 8.0) with 250 mM NaCl (starting buffer) followed by 20 ml of starting buffer containing 12.5 mM imidazole. Protein was eluted from the column using approximately 5 ml of starting buffer with 500 mM imidazole. Fractions containing protein were loaded onto a Hi-Load 26/60 Superdex 200 prep grade gel filtration column (Amersham). Both GFP-AD-Cterm and AD-Cterm-GFP tail fragments elute between 120 ml and 150 ml. GFP-AD elutes between 152 ml and 174 ml. Protein was dialyzed against Mono-Q LSB overnight and then injected onto the Mono-Q HR 5/5 column (Amersham Biosciences) as described above. Protein elutes at about 450 mM NaCl. Protein was used immediately after purification for microscopy and sedimentation assays.

#### Electron microscopy

Purified GFP-tail fragments were diluted to a final protein concentration of approximately 1 μM, a final MgCl_2_ concentration of 10 mM, and a final NaCl concentration of 50 mM. This solution was deposited onto glow-discharged 300-mesh carbon stabilized copper grids coated with formvar (#01753-F; Ted Pella, Redding, California, United States). AD-Cterm and AD proteins were assembled for 2 h, while GFP-containing proteins were assembled for 2 min. A 1% uranyl acetate solution was applied before imaging on a JEOL 1230 transmission electron microscope (JEOL USA, Peabody, Massachusetts, United States).

#### Sedimentation assembly assay

Purified protein was dialyzed overnight against 10 mM imidazole (pH = 7.5), 0.1 mM EDTA, and 1 mM DTT. 10 μM protein was added to an equal volume of 10 mM imidazole, 0.1 mM EDTA, 1 mM DTT, 2× mM NaCl. The final concentration of NaCl was × mM NaCl, where × = 0, 25, 50, 75, 100, 150, and 250 mM. Samples were incubated on ice for at least 30 min and then centrifuged at 132,000 × g for 15 min at 4 °C. Three replicates of each sample were made to ensure reproducibility. Samples of the supernatant and pellet were run on SDS-PAGE gels. The intensity of bands was quantified with an AlphaImager 2000 Documentation and Analysis System (Alpha Innotech Corporation, San Leandro, California, United States).

#### Circular dichroism

An Aviv Circular Dichroism Spectrometer model 62A DS was used (Aviv Corporation, Acton, Massachusetts, United States). The protein was in 10 mM Tris (pH 7.4), 500 mM NaCl, 1 mM EDTA, 1 mM DTT. Wavelength scans were taken from 260 nm to 200 nm. Data were collected every 1 nm with a 1-nm bandwidth and averaging time of 10 s. The spectra presented are buffer subtracted. Thermal melts were taken from 4 °C to 60 °C. θ_222_ was monitored with a 1 nm bandwidth. The temperature was increased in 1 °C increments, and the sample was equilibrated for 2 min at the new temperature before data collection. An averaging time of 30 s was used. The pmt dc voltage used was 1.0 V for 2.5 μM protein and 0.55 V for 50 μM protein. Kaleidagraph (Synergy Software, Essex Junction, Vermont, United States) was used for all data analysis. Plots of fraction denatured versus temperature were constructed as described in Allen and Pielak (1998).

#### Analytical ultracentrifugation

AD-Cterm (3xThr) was assembled by diluting the NaCl concentration to 50 mM. Assembled protein was recovered by centrifugation at 100,000 × g for 15 min. Pellets were resuspended in 10 mM Tris (pH 7.4), 0.1 mM EDTA, 500 mM NaCl (High-Salt Buffer) and centrifuged at 100,000 × g for 15 min to remove aggregates. The AD-Cterm (3xAsp) tail fragment was centrifuged at 100,000 × g for 15 min to remove aggregates. Both tail fragments were exchanged several times into High-Salt Buffer using an Amicon Ultra-15 30,000 Dalton cut-off ultra-filtration device (Millipore, Billerica, Massachusetts, United States). The High-Salt Buffer was the blank in all analytical ultracentrifugation experiments. After buffer exchange, the protein was centrifuged at 14,000 rpm for 10 min in a microcentrifuge to remove aggregates. The protein was snap-frozen in liquid nitrogen and shipped in dry ice to the Macromolecular Interactions Facility at UNC-Chapel Hill. The protein used for all analytical ultracentrifugation experiments was centrifuged at room temperature for 15 min at 16,000 × g to remove aggregates.

All sedimentation equilibrium experiments were performed at the UNC Chapel Hill Macromolecular Interactions Facility as described in Patel et al. (2002). The rotor speed was set at 10,000 rpm, the temperature was maintained at 10 °C, and for the meniscus depletion experiment the conditions were centrifugation at 45,000 rpm for 8 h. AD-Cterm (3xThr) and AD-Cterm (3xAsp) were examined at protein concentrations of 52 μM, 35 μM, 17 μM, and 10.5 μM in High-Salt buffer. The measured densities of the buffers were 1.0 g/ml at 20 °C, and the partial specific volume of both tail fragments was calculated to be 0.73 ml/g at 10 °C (Durschschlag 1986). All data analysis was done using XL-A/XL-I data analysis software version 4.0 (Beckman, Fullerton, California, United States).

#### Culture of *Dictyostelium* cells expressing GFP-RLC-tail fragments

HS1 myosin-null *Dictyostelium* cells (Ruppel et al. 1994) were transformed using electroporation. Cells were grown in 10-cm petri dishes at 22 °C in HL5 medium (Sussman 1987) supplemented with 60 μg/ml penicillin, 60 U/ml streptomycin, and 15 μg/ml G418 (Life Technologies, Carlsbad, California, United States) for plasmid selection.

#### Sedimentation of lysates prepared from *Dictyostelium* cells expressing GFP-RLC-tail fragments

1 ml of nearly confluent *Dictyostelium* cells expressing the appropriate construct was centrifuged and resuspended in 10 mM imidazole (pH 7.5), 0.1 mM EDTA, and 50 mM NaCl in the presence of protease inhibitors. The cells were lysed by freezing in liquid nitrogen and thawing. Cell lysates were allowed to sit on ice for 2 h before centrifugation at 132,000 × g for 20 min. The pellets were resuspended in equal volumes of the lysis buffer. The supernatant and pellets were resolved by SDS-PAGE without boiling the samples. The GFP-fusion proteins were imaged in the gel by excitation at 532 nm on a Typhoon 8000 imager (Molecular Dynamics, Sunnyvale, California, United States).

#### Creation of *Dictyostelium* GFP-myosin expressing stable cell line

The GFP sequence was integrated into the *Dictyostelium* genome upstream of and in-frame with the gene encoding myosin-II *(mhcA)* by homologous recombination. The 167 bp just upstream of *mhcA* through the first 500 bp of the coding region were PCR-amplified from *Dictyostelium* genomic DNA using primers to add a 5′ XhoI site and a 3′ BamHI site. The PCR product was cloned into pBluescript digested with XhoI and BamHI. A PstI site was added between the upstream and *mhcA* coding regions by Quickchange mutagenesis (Stratagene) to create plasmid pBS/upstream-*mhcA*. GFP was amplified from pTX-GFP (Levi et al. 2000) with primers to add 5′ and 3′ PstI sites. The PCR product was then cloned into pBS/upstream-*mhcA* digested with PstI to create pBS/upstream-GFP-*mhcA*. The resulting plasmid was digested with ApaI and XbaI, and the fragment containing GFP flanked by the upstream and coding regions of *mhcA* was gel purified. The fragment was mixed with the blasticidin-resistant plasmid pBsr2 (Sutoh 1993) in a 10:1 excess and transformed into *Dictyostelium*. After the transformed cells were selected with 4 μg/ml blasticidin S (ICN Biochemicals, Costa Mesa, California, United States), a clonal cell line, GMO8B, was created by plating FACS-sorted GFP-positive cells on *Klebsiella* lawns and picking spores from individual GFP-positive plaques. The correct integration of GFP upstream of *mhcA* was verified by PCR and imaging of GFP fluorescence in-gel after SDS-PAGE of cell lysates. The cell line was able to develop normally and grow in suspension, and GFP localization in GMO8B was very similar to GFP-myosin localization in cells expressing GFP-myosin from a plasmid (Moores et al. 1996).

#### Scrape loading of beads into *Dictyostelium* cells

Glass slides were coated with 10 μg/ml polylysine overnight. Approximately 1 ml of semi-confluent GMO8B *Dictyostelium* cells in HL5 medium were allowed to attach to the polylysine-coated slide for 30 min. The medium was then replaced by a 0.04% solution of 0.5 μm-diameter carboxylated red FluoSpheres (Molecular Probes, Eugene, Oregon, United States) in HL5 medium. The cells were immediately scraped off of the surface with a rubber policeman and transferred to a clean glass slide. After the cells were allowed to attach for approximately 30 min, the slide was rinsed in PBS to remove excess beads not taken up by the cells. The cells were then removed from the glass slide by pipetting up and down with HL-5 medium.

#### Live cell fluorescence microscopy

Live cells were transferred to imaging chambers (Applied Scientific, Santa Ana, California, United States) in HL-5 medium. GFP and FluoSphere fluorescence were imaged at room temperature using a Zeiss (Oberkochen, Germany) Axiovert 200 inverted epifluorescence microscope equipped with a 63× objective (N.A. 1.3). Cells were imaged at 20-s intervals. Images were collected using Metamorph (Universal Imaging Corporation, Downington, Pennsylvania, United States) and analyzed with ImageJ (NIH) and Photoshop (Adobe Systems, San Jose, California, United States).

## Abbreviations

3xAla myosin: myosin II with three threonine heavy chain phosphorylation sites at positions 1,823
3xAsp myosin: myosin-II with three threonine heavy chain phosphorylation sites at positions 1,823
a.a.: amino acid(s)
AD: the 34-kDa assembly domain (a.a. 1,531–1,824)
AD-Cterm: the 68-kDa tail fragment starting at the AD and extending to the end of the C terminus of myosin-II (a.a. 1,531–2,116)
BTF: bipolar thick filament
CD: circular dichroism
Cterm: C-terminal domain (a.a. 1,967–2,116)
EM: negative staining electron microscopy
GFP: green fluorescent protein
RLC: regulatory light chain
θ_222_: mean residue ellipticity at 222 nm
