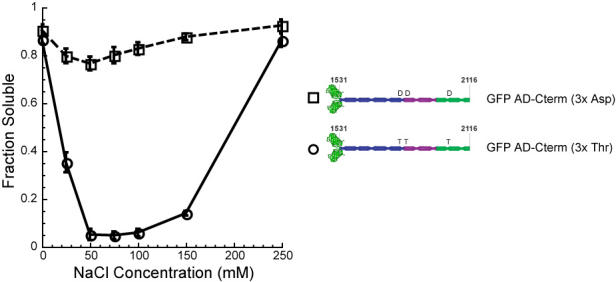# Correction: Dictyostelium Myosin Bipolar Thick Filament Formation: Importance of Charge and Specific Domains of the Myosin Rod

**DOI:** 10.1371/journal.pbio.0030119

**Published:** 2005-03-15

**Authors:** Daniel Hostetter, Sarah Rice, Sara Dean, David Altman, Peggy M McMahon, Shirley Sutton, Ashutosh Tripathy, James A Spudich

10.1371/journal.pbio.0020356

In *PLoS Biology,* volume 2, issue 11.

Figure 3C should have appeared as shown below. The GFP-AD-Cterm (3x Thr) and GFP-AD-Cterm (3x Asp) constructs are slightly less soluble than their headless counterparts. This may be due to the fact that they are somewhat more prone to aggregation over time than the headless proteins. The change does not affect the conclusions of the paper.

**Figure:**